# Pulsed-field gel electrophoresis profile homogeneity of *Mycobacterium avium *subsp. *paratuberculosis *isolates from cattle and heterogeneity of those from sheep and goats

**DOI:** 10.1186/1471-2180-7-18

**Published:** 2007-03-12

**Authors:** Iker Sevilla, Joseba M Garrido, Marivi Geijo, Ramon A Juste

**Affiliations:** 1 Instituto Vasco de Investigación y Desarrollo Agrario (NEIKER), Berreaga, 1, 48160 Derio, Bizkaia, Spain

## Abstract

**Background:**

*Mycobacterium avium *subsp. *paratuberculosis *(*Map*) causes paratuberculosis in animals and is suspected of causing Crohn's Disease in humans. Characterization of strains led to classify paratuberculosis isolates in two main types, cattle type strains, found affecting all host species, and sheep type strains, reported affecting mainly sheep. In order to get a better understanding of the epidemiology of paratuberculosis a large set of *Map *isolates obtained from different species over the last 25 years have been characterized. Five-hundred and twenty isolates from different hosts (cattle, sheep, goats, bison, deer and wild boar) and origins had been cultured and typed by IS*1311 *restriction-endonuclease-analysis. Two-hundred and sixty-nine isolates were further characterized by pulsed-field gel electrophoresis (PFGE) using *Sna*BI and *Spe*I endonucleases. Differences in strain isolation upon various media conditions were also studied.

**Results:**

All bovines, 4 and 26% of Spanish sheep and goats, respectively, and the deer and wild boar studied, carried IS*1311*-Cattle type strains. IS*1311*-Sheep type encompassed 96% and 74% of Spanish sheep and goats, and all three Portuguese sheep. Thirty-seven distinct multiplex PFGE profiles were found, giving 32 novel profiles. Profiles 2-1 and 1-1 accounted for the 85% of cattle isolates. Ten distinct profiles were detected in Spanish sheep, none of them with an incidence higher than 25%. Profile 16-11 (43%) and another three profiles were identified in Spanish caprine cultures. The hierarchical analysis, clustered all profiles found in cattle, "wild" hosts and some small ruminants within the same group. The other group included 11 profiles only found in Spanish sheep and goats, including Spanish pigmented profiles. Differences in growth requirements associated with isolate genotype were observed.

**Conclusion:**

Cattle in Spain are infected with cattle type strains, while sheep and goats are mainly infected with sheep type strains. Although 7H9 broth based culture media seem to broadly cover the growth requirements of most *Map *strains, the use of various solid media is recommended to reduce any recovery biases. High genetic homogeneity of isolates from cattle, and heterogeneity of those from sheep and goats have been detected.

## Background

*Mycobacterium avium *subsp. *paratuberculosis *(*Map*) is an obligate pathogen causing paratuberculosis in ruminants and other animal species, and suspected of being responsible for Crohn's Disease in humans. Paratuberculosis causes significant economic losses to farmers and is becoming prevalent among livestock populations world-wide [1]. Strategies for paratuberculosis prevention and control require an understanding of the epidemiology of the disease. In particular, strain genotyping is a valuable tool for epidemiological tracing of pathogenic micro-organisms.

*Map *isolates are generally identified by acid fast staining, the size of the bacilli, extremely slow growth, dependence on mycobactin, and the presence of several copies of the IS*900 *element in its genome. However, the description of some *Map *strains that are not dependent on mycobactin [2,3] and the presence of IS*900*-like insertion sequences in bacteria other than *Map *[3-6] mean that more reliable methods of characterization would be valuable. The most commonly used typing method is the analysis of restriction fragment length polymorphism using IS*900 *specific probes (IS*900*-RFLP). IS*900*-RFLP classified isolates from cattle, sheep and goats into two major groups using *Bst*EII endonuclease [7]. These were called sheep (*S*) and cattle (*C*) types because of their apparent predominance in those host species. An intermediate (*I*) group was also detected in two sheep from Canada. Strains from sheep are difficult to culture [8,9], making further strain classification difficult. *Bst*EII-*C *or cattle types have been described in cattle from New Zealand, Australia, the USA, Denmark, the Czech Republic, Slovakia, Hungary, Germany, France, Morocco, the United Kingdom, Austria, Italy, the Netherlands, Slovenia, Spain, Sweden, Argentina and Norway [7,10-19]. These RFLP types have also been found infecting sheep from Canada, the USA, the Czech Republic, France, Greece, Australia, Spain and the United Kingdom [7,15-18], and goats from New Zealand, Norway, Argentina, Germany, Denmark, Italy, the United Kingdom, Australia and the USA [7,10-12,16-18]. These strains have also been found in cervids from New Zealand, the Czech Republic, the United Kingdom and Argentina [14,16,20-22], in lagomorphs from Scotland and the Czech Republic [13,21], in alpaca and rhinoceros from Australia [11] and in Czech moufflon and wild boar [21]. In contrast, the literature contains little evidence of *Bst*EII-*S *strains and *Bst*EII-*I *strains being as distributed in animal species: they have been reported in sheep from Canada, New Zealand, the Faroe Islands, South Africa, Morocco and Australia [7,10,11,17,19,23,24], in goats from New Zealand and the Czech Republic [7,15], cattle from the Czech Republic [15], and deer from New Zealand and the Czech Republic [20,21]. IS*1311 *PCR-restriction endonuclease analysis (REA) [25] led to the identification of *S *strains in Australian and Icelandic sheep and cattle [26] and Spanish sheep and goats [27]. A recent study based on IS*1311 *PCR-REA and other two PCR methods reported infection of Spanish goats and bullfighting cattle with *S *(Type I/III) strains [28].

Another promising technique for typing this parasitic bacterium is pulsed-field gel electrophoresis of digested genomic DNA (PFGE). It has been useful for typing other important mycobacteria including *M. avium *and *M. tuberculosis *[29-33]. Pigmented and non-pigmented isolates from a wide variety of hosts and origins were analyzed by *Sna*BI-*Spe*I PFGE in an effort to standardize the technique for paratuberculosis strain typing [34]. Subsequently, caprine isolates from Spain, Scotland and Norway were analyzed [35]. Thus, 26 multiplex profiles have been described and clustered into 3 major types. Other genotyping techniques have been investigated in an attempt to improve upon the discriminatory power of RFLP and PFGE [36-40]. However, some of them do not provide enough additional information and others need to be validated with larger numbers of strains already characterized by RFLP and/or PFGE and from diverse host and geographic origins to assess their value for further epidemiological studies on paratuberculosis.

There have been few studies reporting molecular characterization of Spanish paratuberculosis isolates [27,28,35]. Our aim in this work was to characterize by IS*1311 *PCR-REA and PFGE typing a large set of *Map *isolates obtained at Neiker from different species over the last 25 years to serve as a basis for a better understanding of the epidemiology of paratuberculosis.

## Results

All bovine isolates grew in 2–4 months on HEY, and some cultured after 2002 also grew on LJ or in 7H11 with mycobactin J. Ovine isolates grew in 3–7 months on LJ, 7H11 (with or without mycobactin J) and/or 7H9 (with or without mycobactin J), but only from tissue samples. None of the ovine samples used to inoculate HEY produced *Map *colonies. Goat isolates grew in 2–7 months on the same media as sheep isolates, and some of them were also successfully isolated on HEY. Mycobacterial cells were not successfully propagated from several cases in small ruminants, although the presence of *Map *was identified on the basis of acid fastness, IS*900 *and locus 251 PCR results, and IS*1311 *PCR-REA result. Indian isolates grew in 2–4 months on HEY with no added pyruvate. Deer isolates grew in 3 months on HEY and LJ, and wild boar isolates grew in 2–3 months on HEY and 7H11 with mycobactin J. The isolation media for cultures typed by PFGE are given in Table 1. Subcultures in liquid medium of *C *and *B *strains grew faster than those of *S *strains (data not shown).

### IS*900 *PCR and Locus 251 PCR

PCR amplification detected both IS*900 *and Locus 251 in all 520 isolates used in the study. These findings and the phenotypic characteristics (see "materials and methods" section) allowed all bacterial isolates used to be identified as *Map*.

### IS*1311 *PCR-REA

The IS*1311 *insertion sequence was successfully amplified from all isolates. Restriction analysis of amplicons assigned each isolate to one of the 3 strain types previously described by this method. All bovine isolates, as well as the deer and wild boar isolates, exhibited band patterns corresponding to *C *type strains (Table 2). In contrast, 96.3% and 73.7% of Spanish sheep and goats, respectively, gave isolates with banding patterns corresponding to the *S *type strains; all sheep isolates from Portugal were also classified in this group. The other Spanish sheep (3.7%) and goats (26.3%) were infected by *C *type strains. Interestingly, two goat flocks were infected by both types, some goats carrying a *C *strain and others an *S *strain. All isolates from Indian sheep and goats and from American bison were typed as *B *strains.

### *Sna*BI-*Spe*I PFGE analysis

**Figure 1:**
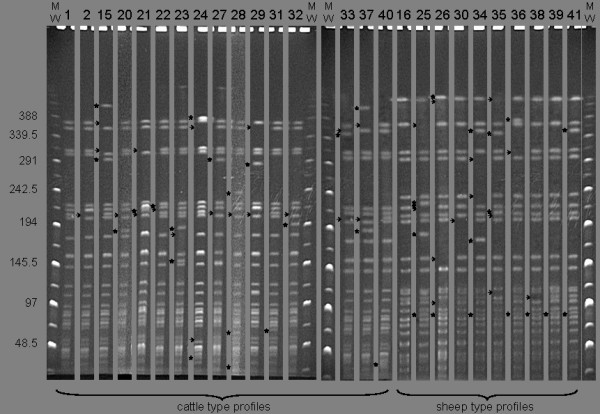
***Sna*BI restriction profiles**. Photographs of two gels comprising the 26 different *Sna*BI pulsotypes detected. Numbers above the lanes correspond to the PFGE nomenclature. Arrows and asterisks indicate differences (band loss and new band, respectively) between type 1 and cattle type profiles, and between type 16 and sheep type profiles. MW = molecular weight standards, in Kilobases (Kb).

**Figure 2:**
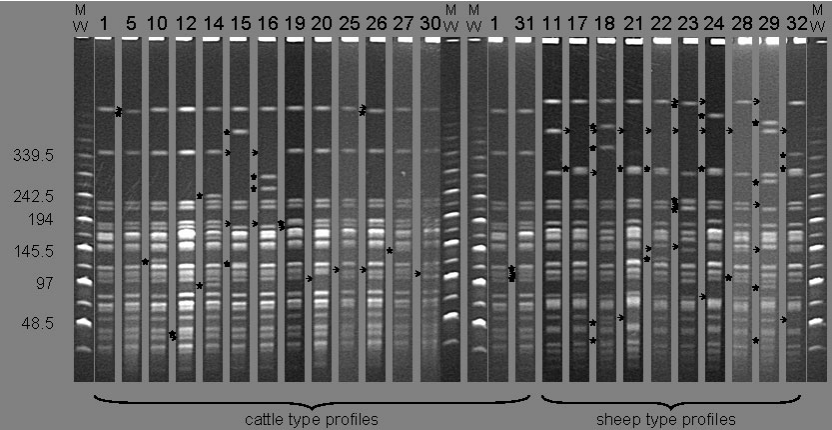
***Spe*I restriction profiles**. Photographs of two gels comprising the 24 different *Spe*I pulsotypes detected. Numbers above the lanes correspond to the PFGE nomenclature. Arrows and asterisks indicate differences (band loss and new band, respectively) between type 1 and cattle type profiles, and between type 11 and sheep type profiles. MW = molecular weight standards, in Kilobases (Kb).

**Figure 3:**
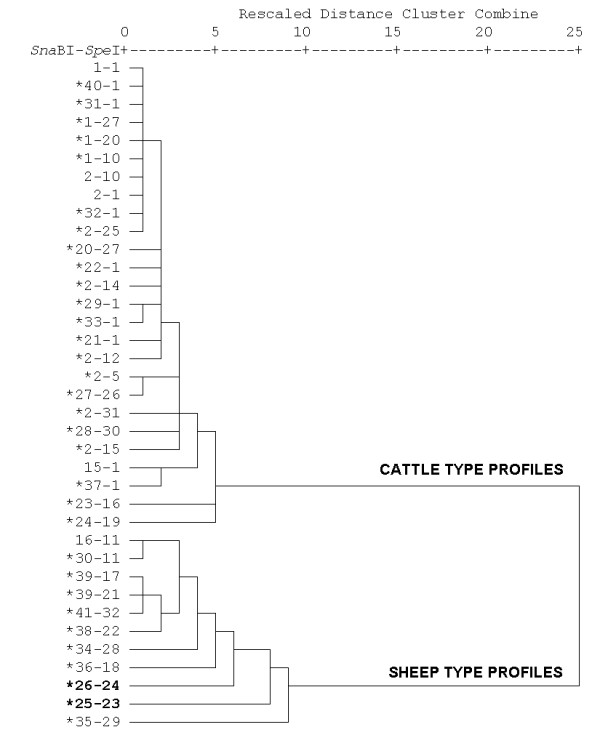
**Dendrogram showing the similarities between multiplex PFGE profiles of isolates in the collection studied**. The agglomerative hierarchical cluster analysis of the data using the unweighted pair group method with arithmetic averages divided strain profiles in two main branches, cattle type profiles and sheep type profiles. Profiles in boldface correspond to pigmented isolates. *novel profiles found in this study.

Restriction endonucleases *Sna*BI and *Spe*I differentiated 26 and 24 pulsotypes, respectively (Figures 1 and 2). Thus, a total of 37 different multiplex PFGE profiles were identified among the 269 *Map *isolates analyzed. Twenty-two of the patterns identified with *Sna*BI (20–41) and 19 of those obtained with *Spe*I (14–32) have not been described previously; the two endonucleases together giving 32 novel PFGE multiplex profiles in our collection of isolates (see Table 1 and Figure 3). Twenty-three different multiplex profiles were detected in Spanish cattle, 10 in Spanish sheep and 4 in Spanish goats. Profiles 2-1 and 1-1 included 52.16 and 32.33% of cattle isolates, found in 58.02 and 30.53% of herds analyzed, respectively. The calculated incidence for each of the other profiles identified in cattle was lower than 1%, except for profile 23-16 which included 3% of bovine isolates. In Spanish sheep, strain type 39-17 was the most frequently found with four isolates detected in 40% of ovine flocks. Four pigmented sheep isolates exhibited novel restriction patterns (26-24 and 25-23), and accounted for the 23.5% of sheep isolates. The other types observed in Spanish sheep appeared only once or twice. Profile 16-11 was found in three goats, and profiles 2-1, 15-1 and 34-28 were identified in a single animal each. The two isolates from deer had profile 37-1, and the wild boar isolate was typed as 2-1. All the Indian isolates gave the same multiplex profile regardless of their host species. All bison isolates from USA were 2-1.

Several herds/flocks carried strains of different profiles: up to 3 in 3 bovine herds and 3 sheep flocks (34 bovine herds, 4 sheep flocks and 1 goat flocks gave more than one isolate). Eleven bovine and one goat herds gave isolates with two different profiles. The other flocks/herds each gave isolates with a single multiplex pattern within the same herd/flock. PFGE typing results according to host species breeds are reported in Table 3.

In five cases, more than one isolate cultured from the same animal was available. Two cows, one goat and one deer yielded two isolates each one but the restriction patterns of isolates from the same animal were identical. In contrast, an isolate cultured in 2002 from the feces of one Holstein bull was of 1-1 type, whereas the two isolates cultured in 2003 from the small intestine and mesenteric lymph nodes of the same animal, were of 2-1 and 28–30 profiles, respectively.

### Phylogenetic analysis of PFGE profiles and estimation of genetic diversity

Phylogenetic analysis of the multiplex profiles obtained produced a dendrogram with two main branches (Figure 3). One includes all profiles found in cattle, deer, wild boar, Indian small ruminants and American bison, for a total of 26 profiles. Previously described profiles 1-1, 2-1, 2–5, 2–10 and 15-1, were part of this group. The other main branch includes 11 profiles only found in sheep and goats from Spain, including the 16-11 profile identified previously and both profiles detected in pigmented isolates (25-23 and 26-24). The discriminatory power for this PFGE strain typing method calculated as Simpson's Index of Diversity, taking into account all isolates and profiles described, was 0.693. The index value was 0.621 for Spanish cattle, and 0.666 for Spanish goats, but much higher, 0.865, for Spanish sheep.

## Discussion

Differences in the success of *Map *isolation and, more specifically, difficulties in culturing this micro-organism from ovine specimens, have been repeatedly reported in many countries (reviewed in reference [9]). Some authors suggest that different host species preferentially carry different strains, and the differing cultivation requirements of these strains causes the divergent efficacy of isolating the organism [2,7,10,41]. In contrast, other researchers did not report any links between host species, strain type and/or culture characteristics [16,17,34]. Our study does not resolve this issue because we did not use the same methods for all cultures, and culture attempts were carried out with the concrete objective of isolating *Map *from paratuberculosis-affected animal specimens received. Nevertheless, our results are consistent with other reports on the comparison of different media for *Map *culture [9,42], and confirm the existence of different culture requirements according to *Map *type. IS*1311 *PCR-REA typing with our collection of bovine isolates – presumably the least biased due to its size and to the use of various culture media – confirms the finding of a previous study [27]: Holstein, Limousin and Pyrenean cattle in Spain are infected with *C *type strains. These strains are more easily isolated on HEY than on other solid media. This strain type was found only in one of 27 isolates from Spanish sheep, the others, like those from Portugal (n = 3) being *S *type. This indicates that sheep in Spain and probably also in Portugal are mostly infected with *S *strains. Some of these were isolated with ease on 7H11 without mycobactin J, others on the same medium with added mycobactin or on LJ with longer incubation periods, and some grew in 7H9 culture flasks (with or without mycobactin J). We did not obtain any growth of such strains on HEY, but not all samples were used to inoculate HEY slants. The single *C *type strain isolated from sheep dates from 1993 and was cultured only on LJ. Consequently, our study does not indicate whether *C *strains can be readily isolated from ovine samples on HEY, but other authors report that this is feasible [7,15]. Choy *et al*. [23] reported that it would appear that the IS*900*-RFLP type is not correlated with the ability to culture isolates, as uncultivable sheep isolates from intestinal-mucosal homogenates in Australia had the same pattern (*S*1) as those from New Zealand grown on HEY [7]. Other factors, including mycobacterial concentration, contamination of inocula, susceptibility to hexadecyl-pyridinium-chloride (or other decontaminants) and/or different phenotypic stages could also contribute to the difficulty of culturing these strains. Only 26% of the Spanish goats analyzed were infected with strains belonging to the *C *type: the other 74% had sheep (*S*) type paratuberculosis. The cultural characteristics of caprine isolates depended on the strain type: *C *strains had bovine-like characteristics and *S *strains had ovine-like characteristics. This strain type distribution in goats disagrees with the findings of other authors in Spain and other countries [12,17,35] who report a very low incidence of *S *strains. The proportion of *S *type isolates previously calculated [28] for Spanish goats (24.7% of goat isolates and 39.1% of flocks studied) is lower than that detected in our study (73.7% of animals and 81.8% of flocks). These differences may be due to a culture media bias, but epidemiological reasons can not be ruled out. We found several goat flocks infected with both *C *and *S *types, although this was not the case for all farms or animal species studied. Our findings suggest that goats are highly susceptible to *C*, *S *and *B Map *strains.

Although we describe numerous multiplex PFGE profiles, nearly the 85% of bovine isolates belonged to only two different profiles, 1-1 and 2-1. The homogeneity of paratuberculosis isolates, especially bovine isolates, has been described before [10-12,19,38,43]. Apparently, particular *Map *strains have developed the ability to infect a wide range of types of cattle, and international trading has favored the worldwide spread of these strains. This may be accentuated by the genetic homogeneity of cattle as a result of genetic improvement programs and international trading. Indeed, bovine genotypes highly susceptible to certain strains may have spread across the world, and consequently the contact between other host species and these strains would have increased proportionally. We noted slight but interesting differences between strain types found in different bovine breeds coming from the same geographic area. Holstein cattle had the largest number of different profiles (n = 18), but this is unsurprising because it was the most numerous breed. More remarkable is the absence from Holsteins of 5 profiles detected in other breeds. Limousine cattle carried more 2-1 type and less 1-1 type than Holstein cattle. In contrast, the 1-1 strain was much more prevalent in Pyrenean cows. Cattle destined to bullfighting came from a less represented geographic area. With the exception of one 2-1 type, strains isolated from these animals (n = 5) showed rare profiles, suggesting a separate epidemiological context. This is in agreement with the results of the study by de Juan *et al*. [28] reporting a high proportion of Type I/III isolates in two bullfighting cattle farms. Type 2–10 detected in 2 bullfighting and 1 Holstein animal, has only previously been reported in one goat from Norway [35]. The novel profile 2–25 was only isolated from two bullfighting animals. The differences of geographic origin of isolates may contribute to the differences found between cattle breeds, especially in bullfighting cattle.

The seven farms affected with 23-16 strains, are all in the same geographic area, some being only 30 km apart, and 5 of them also share the same veterinarian. These strains were isolated over several years (2000–2003) indicating persistence in this ecosystem. According to the phylogenetic analysis, this is one of the strains more different from the others in the bovine type sub-cluster. We are investigating probable links in food and water resources or historic introduction.

Ours is not the first description of mixed infection evidenced by isolation of different strains from the same animal [11,15,19], and could be similar to *Mycobacterium avium *polyclonal infections in human patients [44]. The bull with 3 different strains belonged to a Holstein herd with clinical paratuberculosis cases. The other two isolates obtained from the herd were typed as 1-1 and 2-1. This is consistent with types found in the bull, except for the extra profile (28–30) which was not isolated in any other animal. Re-infection of the animal by different strain types is the most probable explanation, because they were isolated from different biological samples and on different sampling dates.

Strain diversity in the sheep population was high. All the PFGE patterns of Spanish sheep isolates were different from those found in cattle, except for one 2-1 strain from a Rasa-Aragonesa sheep from Aragon. Pigmented isolates exhibited novel restriction patterns designated 26-24 and 25-23. The first was found in Latxa flocks in Basque Country and Navarra. These were not related to the Rasa-Aragonesa sheep from Aragon infected with the other pigmented profile. This is the first report of pigmented strains in Spain. Pigmented strains were less prevalent than the 39-17 strain, found in Basque Country and Navarra. Three flocks in neighboring locations in the main area of prevalence in sheep in Basque Country, gave 3 isolates per flock. For each of the 3 flocks all 3 isolates differed from each other, but 39-17 type was found in all of them.

The diversity index calculated for goats was low. However, including goat isolates reported by de Juan *et al*. [35], the index is 0.817. All isolates we cultured from goats, except one (34-28), have profiles that have previously been reported in this host species. The 16-11 type was observed throughout Spain and in numerous goat breeds. We detected the 15-1 type in goats in Cordoba (Andalucia), and it is also detected in other breeds in Madrid. In conclusion, goats in Spain are affected with a wide range of different strains, including *C *and *S *type strains.

Deer isolates had a novel profile (37-1) closely related to the 15-1 type (see Figure 3), previously found in the same area. Wild boar has carrion-eating behavior, and is a possible reservoir or vector of *Map*, which has been isolated several times from this species [21,45,46]. However, there is no evidence of diseased animals. The strain we isolated from wild boar was 2-1 type, a strain type that is widely disseminated. Cultures from India and bison isolates from USA showed different multiplex profiles, indicating they are different strains.

**Figure 4:**
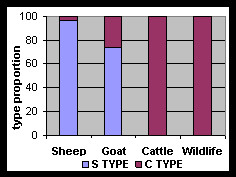
**Strain type polarization in host species**. Graphic representation showing the clear polarization of C and S strain types found in Spanish bovine, ovine, caprine and "wild" species.

The phylogenetic dendrogram we constructed for our collection of paratuberculosis strains shows two distinct branches. One comprises isolates from cattle sheep, goats, deer, wild boar, and bison, whereas the other includes isolates only found in sheep and goats. This is in general agreement with previous work [34,35]. Our first branch (isolates from cattle and other hosts) is the same as the previously described Type II cluster, and IS*900*-RFLP Cattle types. The pigmented isolates from Scotland and Faroe Islands were included in the Type I cluster. However, type 16-11 found later in Spanish goats, was not included in Type I, and they were considered to be Type III. Our pigmented strain profiles clustered together with 16-11 strains and other isolates only found in sheep and goats. It is not clear where these novel profiles should be incorporated, but apparently they are more closely related to the 16-11 profile than to those previously described for pigmented isolates. Other studies included both Sheep and Intermediate IS*900*-RFLP types within the same cluster, composed of two different sub-clusters [11,16]. This seems to be a more accurate approach because Type III strains are very much closer to Type I than to Type II strains. Our findings confirm the soundness of the classification proposed by de Juan *et al*. [28] into 2 major types (Type I/III and Type II). These results suggest, however, that the original classification into cattle (*C*) and sheep (*S*) strains is more descriptive, has historic priority, and is substantially confirmed by the associations between bacterial type and host species (Figure 4). It is also consistent with the widely accepted practice in bacteriology of using as species name the Latin name of the species in which the parasite is found. Consequently, we strongly encourage the use of this system with only two groups corresponding to cattle and sheep.

The overall discriminatory power for this PFGE typing was not high but acceptable (0.693). The problem with this method appears to be the low degree of diversity of the bovine isolates rather than a true lack of power of the typing technique. Multiplex PFGE seems to be one of the most robust and reliable typing methods, because it explores the entire genome and only depends on the specificity of restriction endonucleases used. Other approaches depend on the distribution of repetitive DNA fragments, random amplification of certain fragments or on sequencing of variable repetitive regions; all these approaches can suffer from problems of reproducibility or reliability. However, PFGE is expensive and time consuming, in addition to the problem of obtaining typeable cultures. This method was unable to distinguish the IS*1311*-*B *type found in bison (PFGE 2-1) from other IS*1311*-*C *strains. The discrimination between IS*1311*-*B *and *C *is important, because these *B *strains (from both India and USA) share the phenotypic characteristic of preferring egg-based media without added pyruvate [27].

## Conclusion

Improved liquid culture media (based on Middebrook 7H9) seem to be appropriate for primary isolation, because they appear to provide the entire isolation requirement spectrum of all strains. Nevertheless, we recommend the use of various solid media to avoid biases in primary isolation, and subculture of liquid cultures (when used for primary isolation) onto solid media to obtain pure colonies. Alternatively, PCR-based differentiation of strain type before culture inoculation may help to decide the media to be used.

High homogeneity of isolates from cattle, and heterogeneity of those from sheep and goats have been detected. Overall typing may need to be improved and this could be done by including an additional endonuclease or complementary techniques. PCR-REA typing is a powerful technique because it can both confirm *Map *identity and provide clear microbiological and epidemiological information. Systematic use of the more sophisticated PFGE method does not seem to be of much more benefit: it provides genetic information which does not appear to be clearly related to phenotypic properties but rather to limited epidemiologic details.

## Methods

### Sources and growth of isolates

**Figure 5:**
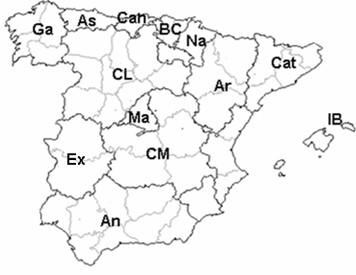
**Map of Spain**. Regions mentioned in the study are indicated as follows: An = Andalucia; Ar = Aragón; As = Asturias; BC = Basque Country; Can = Cantabria; Cat = Cataluña; CL = Castilla y León; CM = Castilla-La Mancha; Ex = Extremadura; Ga = Galicia; IB = Balearic Islands; Ma = Madrid; Na = Navarra.

**Figure 6:**
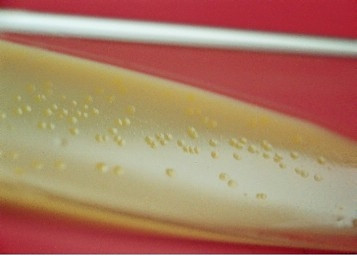
**Pigmented ovine isolate**. Pigmented colonies of *Mycobacterium avium *subsp. *paratuberculosis *on a Lowënstein-Jensen slant, isolated from the yellow-colored intestines of one sheep with clinical paratuberculosis from the Basque Country (Spain).

Feces and tissue samples from cattle, sheep and goats were collected for routine paratuberculosis confirmation and for research purposes between 2000 and 2005. Twelve samples from sheep and one from a goat date from 1984–1999, and isolates obtained from these samples were maintained as glycerol stocks at -80°C. Most strains were from animals in northern regions of Spain, but strains from other Spanish regions were also included (see Figure 5). Four-hundred and forty-four paratuberculosis isolates were recovered from cattle (n = 409; 178 herds), 27 from sheep (n = 27; 15 flocks) and 21 from goats (n = 19; 11 flocks). Four of the ovine isolates were yellow pigmented (Figure 6). *Map *was also cultured from one deer (2 isolates from 2 different tissue samples) and one wild boar. Eight bovine cultures from Argentina, one from France and 3 ovine cultures from Portugal were also included, as well as isolates from American bison (n = 3) and Indian sheep (n = 2) and goats (n = 8). Samples were processed for culture of *Map *as described elsewhere [2]. Bovine samples were cultured on Herrold's egg yolk medium supplemented with mycobactin J (2 mg/l in all media containing the mycobactin) (Allied Monitor, Inc., Fayette, MO, USA) and sodium pyruvate (HEY). To avoid any bias in the recovery of strains from cattle, additional tubes of mycobactin J-supplemented Lowënstein-Jensen medium (LJ) were inoculated with bovine samples collected after 2002. Some bovine samples were also used to inoculate Middlebrook 7H11 supplemented with 1% Middlebrook OADC Enrichment (Becton, Dickinson and Company, MD, USA) (7H11) and mycobactin J. Ovine and caprine samples were cultured on HEY, on LJ and/or on 7H11 (alternatively with mycobactin J), depending on when they were collected. Some of these cultures failed in the first attempt, and material stored at -20°C was subsequently cultured again on Middlebrook 7H9 broth supplemented with OADC, 0.05% Tween 80 (Panreac Quimica SA, Barcelona, Spain) (7H9) with or without added mycobactin J, and then sub-cultured on 7H11. Indian samples were initially grown on HEY without added pyruvate and bison samples on LJ. Deer samples were cultured on HEY and LJ, and wild boar samples on these two media and on 7H11 with mycobactin J.

### Cultures for PFGE

High quality DNA could not be prepared from all samples due to loss of viability of some of the older isolates. Sufficient growth was obtained for PFGE typing with 232 isolates from cattle (n = 228; 131 herds), 17 isolates from sheep (n = 17; 10 flocks), seven isolates from goats (n = 6; 5 flocks), two from deer, one from wild boar, three from American bison and seven from Indian small ruminants (two from sheep and five from goats). Nearly 85% of the bovine isolates were from Holstein (dairy) cattle, 10% from Limousin (beef) cows, 3% from Pyrenean (beef) cows, and 2% from Lidia (bullfighting) cattle. Spanish sheep were Latxa (dairy; n = 14), Rasa-Aragonesa (meat; n = 2) and Carranzana (dairy; n = 1). Goat breeds were Payoya (dairy/meat; n = 3), Murciano-Granadina (dairy/meat; n = 3) and Saanen (dairy; n = 1). The Indian sheep (Muzzafarnagari) and goats (Barbari) were all meat breeds.

### Isolate identification: IS*900 *PCR and Locus 251 PCR

*Map *was identified on the basis of time of incubation to visible colonies, colony and bacillus morphology including acid fastness, and mycobactin dependence on egg-based media. Isolates were screened for the presence of IS*900 *and Locus 251 [47] as follows. The method described by Garrido *et al*. [48] was used to extract DNA from single colonies or if there was no visible growth on solid media from broth culture pellets obtained by centrifugation of 5 ml at 2000 × g for 15 min. A GeneAmp 9600 PCR system (Applied Biosystems, Foster City, CA, USA) with specific primers were used for amplification under standardized conditions as described previously [47,48]. PCR products were subjected to electrophoresis in 2% (w/v) agarose gels and stained with ethidium bromide to reveal DNA bands.

### IS*1311 *PCR-REA

A segment of IS*1311 *element was amplified and digested with *Hinf*I and *Mse*I (*Mse*I only to rule out presence of *Mycobacterium avium *subsp. *avium*) endonucleases as described by Marsh *et al*. [25]. DNA fragments were separated by electrophoresis in 4% (w/v) agarose gels stained with ethidium bromide. Isolates were classified as cattle (*C*), sheep (*S*) or bison (*B*) *Map *types according to previously published criteria [25,49].

### *Sna*BI-*Spe*I PFGE analysis

A protocol described previously [34] was used to prepare mycobacterial DNA. Ten ml of 7H9 broth was inoculated and incubated at 37°C in a static 25 cm^3 ^cell culture flask (Corning Inc., Corning, NY, USA). When sufficient growth was observed at the bottom of the flask, the culture was homogenized by vigorous shaking and the optical density measured using a Densimat (Bio-Mérieux, Marcy L'Etoile, France). An appropriate volume of culture was centrifuged at 2000 × g for 15 min, and the pellet washed and resuspended in modified spheroplasting buffer to obtain a bacterial suspension of approximately 6 × 10^9 ^bacteria/ml. This suspension was heated to 55°C and mixed with an equal volume of molten 1.5% (w/v) low-melting-point agarose (Biorad, Hemel Hempstead, Hertfordshire, United Kingdom) in 50 mM EDTA, then poured into plug molds (Biorad), and allowed to solidify at 4°C. The plugs were incubated in a Tris-EDTA (10 mM Tris-HCl and 1 mM EDTA; pH 8) lysis solution containing 1.5 mg/ml lysozyme at 37°C for 16–20 h. They were then placed with ESP solution (0.5 M EDTA, 1% (w/v) lauryl sarcosine and 1.5 mg/ml proteinase K) and incubated at 55°C for at least 3 days. The ESP solution was discarded and the agarose plugs were washed in pH 8 Tris-EDTA 6 times for 20 min each. The plugs were equilibrated in the restriction buffer supplied by the enzyme manufacturer (1 × NE buffer and 0.1 mg/ml bovine serum albumin; New England Biolabs, Inc., Beverly, MA, USA) and then subjected to digestion with 20 U of the appropriate endonuclease in fresh buffer at 37°C (*Sna*BI, 3 h; *Spe*I, overnight). The plugs were placed in pH 8 Tris-EDTA and loaded into a 1% (w/v) pulsed field certified agarose gel (Biorad). Lambda midrange II PFG markers (New England Biolabs) and the type strain ATCC 19698 were included in every gel. Electrophoresis was carried out in a CHEF-DRII apparatus (BioRad). *Sna*BI restricted DNA was separated with initial and final switches of 6.8 and 26.3, respectively, and for separation of *Spe*I digests the values were 2.2 and 35.4, respectively. The electrophoresis time for *Sna*BI was 28 h to allow rapid screening of isolates, and samples with different and/or doubtful patterns were then electrophoresed for 40 h under the same conditions. Electrophoresis for *Spe*I gels was for 23 h. The gradient applied was always 6 V/cm. DNA bands were visualized and photographed in a Fluor-S MultiImager (BioRad) after ethidium bromide staining. Nomenclature of novel patterns was as recommended elsewhere [34,35]. The first number refers to the *Sna*BI profile and the second to the *Spe*I profile, giving consecutive numbers to new profiles. Both molecular size markers and ATCC 19698 *Sna*BI and *Spe*I profiles were used as between-gel references for band identification.

### Phylogenetic analysis of PFGE profiles and estimation of genetic diversity

PFGE images were analyzed with the Quantity One Software package version 4.5 (BioRad). Similarities between different multiplex profiles were calculated as Jaccard coefficients on the basis of band presence/absence scores with the SPSS software for Windows version 11.0 (SPSS Inc., Chicago, IL, USA). An agglomerative hierarchical cluster analysis of the data using the unweighted pair group method with arithmetic averages was used to construct a dendrogram. Simpson's Index of Diversity was calculated as follows to compare the genetic diversity of the isolates between host species and to asses the discriminatory power of the *Sna*BI-*Spe*I-PFGE typing: 1-[Σ(no. of isolates with a particular multiplex profile/total no. of isolates)^2^]

## Authors' contributions

IS carried out the laboratory and field work, compiled and analysed information and data, and drafted the manuscript as part of his PhD dissertation. JMG participated in the laboratory and field work, compiled information, coordinated the study and helped to draft the manuscript. MG participated in the laboratory and field work. RAJ conceived and coordinated the study, and helped to draft the manuscript. All authors read and approved the final manuscript.

## Figures and Tables

**Table 1:** *Sna*BI-*Spe*I PFGE classification of *Map *strains.

Country	PFGE profile	Locations	Host sp	No. of isolates (%)	No. of herds or flocks (%)	Other types in same herd/flock (No. of herds/flocks)	Primary Isolation	IS*1311 *type
Spain	1-1	BC, As, Ar, Na, Cat	Cattle	75 (32.33)	40 (30.53)	1–20 (1), 2-1 (6), 22-1 (1), 23-16 (1), 28–30 (1), 31-1 (1)	HEY, LJ, 7H11*	*C *type
	*1–10	BC	Cattle	1 (0.43)	1 (0.76)		HEY	*C *type
	*1–20	Na	Cattle	1 (0.43)	1 (0.76)	1-1 (1), 31-1 (1)	HEY	*C *type
	*1–27	BC	Cattle	3 (1.29)	1 (0.76)		HEY	*C *type
	2-1	BC, As, Can, Ga, CL, Ar, Na, Cat, CM	Cattle	121 (52.16)	76 (58.02)	1-1 (6), 2–10 (2), 2–15 (1), 2–31 (1), 22-1 (1), 23-16 (2), 28–30 (1)	HEY, LJ, 7H11*	*C *type
	2–5	As	Cattle	1 (0.43)	1 (0.76)		HEY	*C *type
	2–10	Can, CL, Ex	Cattle	3 (1.29)	3 (2.29)	2-1 (2)	HEY	*C *type
	*2–12	BC	Cattle	1 (0.43)	1 (0.76)		HEY	*C *type
	*2–14	Ga	Cattle	1 (0.43)	1 (0.76)		HEY	*C *type
	*2–15	BC, Ma	Cattle	3 (1.29)	3 (2.29)	2-1 (1), 23-16 (1)	HEY	*C *type
	*2–25	CL	Cattle	2 (0.86)	1 (0.76)		HEY	*C *type
	*2–31	CL	Cattle	1 (0.43)	1 (0.76)	2-1 (1)	HEY	*C *type
	*20–27	BC	Cattle	2 (0.86)	2 (2.29)		HEY	*C *type
	*21-1	Can	Cattle	1 (0.43)	1 (0.76)		HEY	*C *type
	*22-1	Ar	Cattle	1 (0.43)	1 (0.76)	1-1 (1), 2-1 (1)	HEY	*C *type
	*23-16	BC	Cattle	7 (3.02)	7 (5.34)	1-1 (1), 2-1 (2), 2–15 (1)	HEY	*C *type
	*24-19	Ma	Cattle	1 (0.43)	1 (0.76)		HEY	*C *type
	*27-26	BC	Cattle	1 (0.43)	1 (0.76)		HEY	*C *type
	*28–30	BC	Cattle	1 (0.43)	1 (0.76)	1-1 (1), 2-1 (1)	HEY	*C *type
	*31-1	Na	Cattle	1 (0.43)	1 (0.76)	1-1 (1), 1–20 (1)	HEY	*C *type
	*32-1	BC, Na	Cattle	2 (0.86)	2 (1.53)		HEY	*C *type
	*33-1	BC	Cattle	1 (0.43)	1 (0.76)		HEY	*C *type
	*40-1	CL	Cattle	1 (0.43)	1 (0.76)		HEY	*C *type
	2-1	Ar	Sheep	1 (5.88)	1 (10.0)		LJ	*C *type
	*25-23	Ar	Sheep	1 (5.88)	1 (10.0)		LJ	*S *type
	*26-24	BC, Na	Sheep	3 (17.65)	2 (20.0)		LJ, 7H9*+7H11	*S *type
	*30-11	BC	Sheep	2 (11.76)	2 (20.0)	39-17 (2), 39-21 (1), 41-32 (1)	7H11	*S *type
	*35-29	BC	Sheep	1 (5.88)	1 (10.0)		7H11*, 7H9+7H11	*S *type
	*36-18	BC	Sheep	1 (5.88)	1 (10.0)	38-22 (1), 39-17 (1)	7H11	*S *type
	*38-22	BC	Sheep	1 (5.88)	1 (10.0)	36-18 (1), 39-17 (1)	7H11	*S *type
	*39-17	BC, Na	Sheep	4 (23.53)	4 (40.0)	30-11 (2), 36-18 (1), 38-22 (1), 39-21 (1), 41-32 (1)	7H11	*S *type
	*39-21	BC	Sheep	2 (11.76)	2 (20.0)	30-11 (1), 39-17 (1)	7H11	*S *type
	*41-32	BC	Sheep	1 (5.88)	1 (10.0)	30-11 (1), 39-17 (1)	7H11	*S *type
	2-1	BC	Goat	2 (28.57)	1 (20.0)		HEY, 7H11*	*C *type
	15-1	An	Goat	1 (14.29)	1 (20.0)	34-28 (1)	7H11*, 7H9+7H11	*C *type
	16-11	CL, An, IB	Goat	3 (42.86)	3 (60.0)		LJ, 7H9+7H11	*S *type
	*34-28	An	Goat	1 (14.29)	1 (20.0)	15-1 (1)	7H9+7H11	*S *type
	*37-1	CM	Deer	2 (100.0)	1 (100.0)		HEY	*C *type
	2-1	CM	Wild Boar	1 (100.0)	1 (100.0)		HEY, 7H11*	*C *type

India	*29-1	Mathura	Sheep	2 (100.0)	1 (100.0)		HEY	*B *type
	*29-1	Farah	Goat	5 (100.0)	1 (100.0)		HEY	*B *type

USA	2-1	Montana	Bison	3 (100.0)	1 (100.0)		LJ	*B *type

Details of SnaBI-SpeI multiplex profiles of the 269 Map isolates studied by PFGE: geographic origin, number of isolates, number of herds/flocks of the indicated host species, primary isolation media and IS1311 PCR-REA type. Profiles in boldface correspond to pigmented isolates. *novel profiles found in this study. Abbreviations: An = Andalucía; Ar = Aragón; As = Asturias; BC = Basque Country; Can = Cantabria; Cat = Cataluña; CL = Castilla y León; CM = Castilla-La Mancha; Ex = Extremadura; Ga = Galicia; IB = Balearic Islands; Ma = Madrid; Na = Navarra. HEY = Herrold's Egg Yolk Medium with mycobactin J; LJ = Löwenstein-Jensen Medium with mycobactin J; 7H11 = OADC supplemented Middlebrook 7H11 Medium without mycobactin J; 7H11* = OADC supplemented Middlebrook 7H11 Medium with mycobactin J; 7H9+7H11 = OADC supplemented Middlebrook 7H9 Broth without mycobactin J, subcultured on 7H11; 7H9*+7H11 = OADC supplemented Middlebrook 7H9 Broth with mycobactin J, subcultured on 7H11.

**Table 2:** IS*1311 *classification of *Map *strains.

Geographic origin		Host sp.	no. of isolates	no. of animals(%)	no. of herds/flocks(%)	IS*1311 *type
Spain	• Ar (19), As (9), BC (228), Can (22), CL (92), CM (2), Cat (9), Ex (1), Ga (13), Ma (4), Na (46)	Cattle	444	409 (100.0)	178 (100.0)	*C*
	• Ar	Sheep	1	1 (3.7)	1 (6.67)	*C*
	• An (1), Ar (2), BC (17), Na (5)	Sheep	26	26 (96.3)	14 (93.33)	*S*
	• An (4), BC (3; 1 goat)	Goat	7	5 (26.32)	4 (36.36)	*C*
	• An (11), CL (1), Ga (1), IB (1)	Goat	14	14 (73.68)	9 (81.82)	*S*
	• CM	Deer	2	2 (100.0)	1 (100.0)	*C*
	• CM	Wild boar	1	1 (100.0)	1 (100.0)	*C*
Argentina	--	Cattle	8	8 (100.0)	-	*C*
France	• Lys	Cattle	1	1 (100.0)	1 (100.0)	*C*
Portugal	• Trás-os-Montes	Sheep	3	3 (100.0)	2 (100.0)	*S*
India	• Mathura	Sheep	2	2 (100.0)	1 (100.0)	*B*
	• Farah	Goat	8	8 (100.0)	1 (100.0)	*B*
USA	• Montana	Bison	3	3 (100.0)	1 (100.0)	*B*

		Total	520	483		

The 520 Map isolates from different host species classified as C (cattle), S (sheep) or B (bison) type according to IS1311 PCR-REA. Numbers of isolates from particular regions in Spain are specified in brackets following the region abbreviation. Abbreviations: An = Andalucía; Ar = Aragón; As = Asturias; BC = Basque Country; Can = Cantabria; Cat = Cataluña; CL = Castilla y León; CM = Castilla-La Mancha; Ex = Extremadura; Ga = Galicia; IB = Balearic Islands; Ma = Madrid; Na = Navarra.

**Table 3:** Distribution of *Map *genotypes among the different breeds of host species.

	CATTLE (232 isolates & 228 animals)				SHEEP (17 isolates & 17 animals)			GOAT (7 isolates & 6 animals)		
Breed	Holstein	Limousin	Pyrenean	Bullfight	Latxa	Rasa-Aragonesa	Carranzana	Payoya	Murciano-Granadina	Saanen
Isolates	197 (84.91)	23 (9.91)	7 (3.02)	5 (2.16)	14 (82.35)	2 (11.76)	1 (5.89)	3 (42.86)	3 (42.86)	1 (14.29)
Animals	193 (84.65)	23 (10.09)	7 (3.07)	5 (2.19)	14 (82.35)	2 (11.76)	1 (5.89)	3 (50.0)	2 (33.33)	1 (16.67)
Herds/Flocks	100 (76.34)	22 (16.79)	6 (4.58)	3 (2.29)	7 (70.0)	2 (20.0)	1 (10.0)	2 (40.0)	2 (40.0)	1 (20.0)

1-1/*C*	68 (34.52)	3 (13.04)	4 (57.14)							
1–10/*C*			1 (14.29)							
1–20/*C*	1 (0.51)									
1–27/*C*	3 (1.52)									
2-1/*C*	103 (52.28)	15 (65.22)	2 (28.57)	1 (20.0)		1 (50.0)			2 (66.67)	
2–5/*C*	1 (0.51)									
2–10/*C*	1 (0.51)			2 (40.0)						
2–12/*C*	1 (0.51)									
2–14/*C*	1 (0.51)									
2–15/*C*	3 (1.52)									
2–25/*C*				2 (40.0)						
2–31/*C*	1 (0.51)									
15-1/*C*								1 (33.33)		
20–27/*C*		2 (8.70)								
21-1/*C*	1 (0.51)									
22-1/*C*	1 (0.51)									
23-16/*C*	6 (3.05)	1 (4.35)								
24-19/*C*		1 (4.35)								
27-26/*C*	1 (0.51)									
28–30/*C*	1 (0.51)									
31-1/*C*	1 (0.51)									
32-1/*C*	2 (1.02)									
33-1/*C*		1 (4.35)								
40-1/*C*	1 (0.51)									

16-11/*S*								1 (33.33)	1 (33.33)	1 (100.0)
25-23/*S*						1 (50.0)				
26-24/*S*					3 (21.43)					
30-11/*S*					2 (14.29)					
34-28/*S*								1 (33.33)		
35-29/*S*							1 (100.0)			
36-18/*S*					1 (7.14)					
38-22/*S*					1 (7.14)					
39-17/*S*					4 (28.57)					
39-21/*S*					2 (14.29)					
41-32/*S*					1 (7.14)					

Number of isolates, animals and farms in Spain giving isolates of each SnaBI-SpeI multiplex profile/IS1311 type according to the host species breed. Percentages are in brackets.
